# Does Vitamin D3 supplementation improve Depression scores among rural adolescent? A Cluster Randomized control trial

**DOI:** 10.1192/j.eurpsy.2023.1559

**Published:** 2023-07-19

**Authors:** P. T. Satyanarayana, R. Suryanarayana, S. TY, S. Reddy, N. AG

**Affiliations:** 1 Community Medicine; 2 Biochemistry; 3 Pediatrics; 4 Psychiatry, SDUMC, SDUAHER, Kolar, Karanataka, India, KOlar, India

## Abstract

**Introduction:**

Contemporary evidence has established that stunted vitamin D levels are associated with depression, poor mood and other mental disorders.Vitamin D supplementation might play a significant role in depression.

**Objectives:**

To assess the role of supplementation of Vitamin D on depression scores among rural adolescents

**Methods:**

It is a Cluster Randomized Control Trial carried out for a period of two years. 10 Government rural schools were taken as clusters and sample size was calculated using previous literature.(Libuda.et al:EJN. 2020; 27:1-0) 150 was the final sample size in each group. Adolescents aged 11-18 years were included and with any pre-existing mental health illness, renal abnormalities and confirmed neurological disorders (Epilepsy) were excluded. Intervention group received 2 months of 2000 IU per day for 9 weeks and Control arm received 500 mg of Calcium and low dose (250 IU) of Vitamin D. To assess socio-demographic status a pretested semi structured questionnaire was used. To assess depression, Becks Depression Inventory (BDI-II) was used. Venous Blood was taken by experienced lab technician and analyzed in Central Diagnostic Laboratory Services, Biochemistry Department, SDUMC, SDUAHER, Kolar, Karnataka, India. Study was started after Central ethics Committee approval. This study is intramural funded project (Rs.6,12,000/). Clinical Trials Registry number: CTRI/2021/07/034654: REF/2021/03/042355. All the data collected entered in Microsoft excel and analysed using SPSS v 22(IBM corp,USA). Pre-intervention and post intervention difference assessed with t-test summarized as Mean and Standard deviation (SD) with statistically significant difference defined with p value less than 0.05. Both Intention-To-Treat analysis and Per Protocol analysis done and reported separately.

**Results:**

**Table:** 

Distribution of adolescent school children according to sociodemographic profile	Vitamin D supplementation arm (n=235)	Calcium supplementation Arm (n=216)			
	Frequency	Percent	Frequency	Percent	
Age in years	14	8	3.4	42	19.4
	15	129	54.9	143	66.2
	16	86	36.6	31	14.4
	17	12	5.1	00	00
Gender	Boys	124	52.8	100	46.3
	Girls	111	47.2	116	53.7
Type of Family	Nuclear	187	79.6	136	63.0
	Joint	48	20.4	80	37.0

Comparing Becks Depression scores before and after intervention, Vitamin D arm showed statistically significant reduction in Becks Depression scores. Intention to treat analysis showed that Vitamin D arm had statistically significant reduction in Becks Depression scores.

**Conclusions:**

Vitamin D supplementation had reduced Depression scores among rural adolescents significantly. Vitamin D toxicity was not noted inspite of High dose of Vitamin D supplementation

**Disclosure of Interest:**

P. Satyanarayana Grant / Research support from: SDUAHER Intramural research , R. Suryanarayana: None Declared, S. TY: None Declared, S. Reddy: None Declared, N. AG: None Declared

